# Epidemiology of Recovery From Alcohol Use Disorder

**DOI:** 10.35946/arcr.v40.3.02

**Published:** 2020-11-12

**Authors:** Jalie A. Tucker, Susan D. Chandler, Katie Witkiewitz

**Affiliations:** 1 Department of Health Education and Behavior and the Center for Behavioral Economic Health Research, University of Florida, Gainesville, Florida; 2 Department of Psychology and the Center on Alcoholism, Substance Abuse, and Addictions, University of New Mexico, Albuquerque, New Mexico

**Keywords:** alcohol, alcohol use disorder, recovery, remission, natural recovery, epidemiology, alcohol treatment utilization, low-risk drinking

## Abstract

Almost one-third of the U.S. population meets alcohol use disorder (AUD) criteria on a lifetime basis. This review provides an overview of recent research on the prevalence and patterns of alcohol-related improvement and selectively reviews nationally representative surveys and studies that followed risk groups longitudinally with a goal of informing patients with AUD and AUD researchers, clinicians, and policy-makers about patterns of improvement in the population. Based on the research, alcohol use increases during adolescence and early adulthood and then decreases beginning in the mid-20s across the adult life span. Approximately 70% of persons with AUD and alcohol problems improve without interventions (natural recovery), and fewer than 25% utilize alcohol-focused services. Low-risk drinking is a more common outcome in untreated samples, in part because seeking treatment is associated with higher problem severity. Sex differences are more apparent in help-seeking than recovery patterns, and women have lower help-seeking rates than men. Whites are proportionately more likely to utilize services than are Blacks and Hispanics. Improving recovery rates will likely require offering interventions outside of the health care sector to affected communities and utilizing social networks and public health tools to close the longstanding gap between need and utilization of AUD-focused services.

## INTRODUCTION

Substance use disorder (SUD) is among the most prevalent mental health disorders in the United States and in general clinical practice, with 7% of the U.S. population age 12 and older (19.7 million people) having an SUD of some kind in 2018.^1^ Alcohol use disorder (AUD) is the most prevalent SUD, with 5% of persons age 12 and older reporting AUD in 2018.^1^ Of persons with an SUD in 2018, and excluding those with a tobacco use disorder, 60% had AUD, 27% had an illicit drug use disorder, and 13% had disorders involving alcohol and illicit drugs.^1^ On a lifetime basis, almost one-third of persons in the United States meet criteria for AUD.^2^ In addition to the high AUD prevalence, many more individuals engage in risky drinking or experience alcohol-related negative consequences that fall short of meeting clinical diagnostic criteria for AUD.^3^ Thus, harmful alcohol use is a major public health problem, costing the United States approximately $250 billion per year, and it is the third leading cause of preventable death.^4^

Most individuals who develop an AUD or have subclinical alcohol-related problems will reduce or resolve their problem on their own or with assistance from professional alcohol treatment or mutual help groups.^5^–^9^ The epidemiology of this robust phenomenon is the focus of this article. After initial consideration of complexities involved in defining improvement in alcohol-related problems, which is discussed in depth by Witkiewitz et al.,^10^ this article describes the prevalence and heterogeneity of pathways to recovery and examines relationships between patterns of seeking help for and improvements in alcohol-related problems. Then, the topic is examined from a life span developmental perspective, which is less well-researched and involves relationships among age-related rates of problem onset, reduction, and persistence. The final section discusses differences in the overall patterns previously discussed as a function of gender and race/ethnicity. Emphasis is placed on illustrative recent findings. Earlier work is covered in prior literature.^11^,^12^

## DEFINING IMPROVEMENT IN ALCOHOL-RELATED PROBLEMS

As discussed by Witkiewitz et al.,^10^ the conceptualization and measurement of improvements among persons with AUD and the constellation of improvements that define “recovery” have been debated for decades and continue to evolve. Clinical diagnostic criteria for AUD are offered by the American Psychiatric Association’s fifth edition of the *Diagnostic and Statistical Manual of Mental Disorders* (DSM-5)^3^ and the World Health Organization,^13^ with the former predominating in the United States. Numerous reputable organizations offer definitions of low- and high-risk drinking practices^4^,^14^ as well as AUD recovery or remission.^15^ These various criteria have been revised over time as research evidence has accumulated, generally in the direction of recognizing that alcohol consumption and AUD occur on severity continua. Furthermore, most individuals who engage in harmful alcohol use either do not meet AUD criteria or meet criteria for a mild disorder characterized by lower levels of symptomology.^16^

Characterizations of improvement in alcohol-related problems have correspondingly become more nuanced over time in recognition of the heterogeneity of pathways, processes, and outcomes relevant to understanding how people reduce or resolve alcohol-related problems.^10^ The term “recovery” is generally reserved for broad-based, sustained improvements in drinking practices and other areas of functioning adversely affected by drinking. Therefore, this article uses the term “recovery” to refer to a broadly conceived process resulting in sustained improvements in multiple domains, and uses the term “remission” to refer to more limited improvements in specific symptoms or problem behaviors (e.g., drinking practices). This is in line with the National Institute on Alcohol Abuse and Alcoholism’s (NIAAA) recent definition of recovery from AUD as distinct from remission from AUD, defined symptomatically based on DSM-5 criteria, or cessation of heavy drinking without characterizing the presence or absence of other symptoms or improvements. It also is consistent with other recovery definitions, including those from the recovery community or patient perspectives, that encompass improved well-being and functioning and are not limited to attainment of abstinence or stable low-risk drinking.^8^,^17^

It is also important to acknowledge the association of the term “recovery” with Alcoholics Anonymous (AA) and other mutual support groups. Even though the term is widely used in the clinical literature, many persons attempting to resolve their alcohol problems do not identify with being in recovery^8^ and reject clinical labels indicative of AUD, especially those individuals attempting to resolve a drinking problem on their own.^9^ Moreover, salutary improvements can occur in circumscribed areas of alcohol-related dysfunction, and reductions in drinking can contribute to improved health and well-being even if ongoing drinking falls short of traditional definitions of recovery that emphasize abstinence as a required element.^18^

As discussed by Witkiewitz and Tucker,^16^ a core issue debated for decades is the extent to which drinking practices should be central to defining improvement or recovery. Early writings regarded sustained abstinence as the hallmark of recovery among persons with severe alcohol problems who had repeatedly been unable to limit their drinking or abstain.^19^ Newer clinical diagnostic systems such as DSM-5 emphasize development of tolerance and physical dependence and drinking in harmful ways and under conditions that increase risk for adverse consequences.^3^ Drinking practices are not a criterion in accepted diagnostic systems for AUD, including DSM-5, and most schemes define recovery based on symptom reduction, improved functioning, and well-being and are not heavily focused on drinking practices per se. Yet, the large treatment outcome literature concerned with promoting recovery has relied heavily on drinking practices as the major outcome metric, typically by using quantity-frequency criteria considered indicative of higher-risk drinking practices (any occasions of more than 14 drinks weekly or more than five drinks daily for men; more than seven drinks weekly or more than four drinks daily for women in the past year).^4^,^14^

Recent work, however, has shown that such consumption-based thresholds lack sensitivity and specificity for predicting problems related to drinking and do not differentiate individuals based on measures of health, functioning, and well-being.^20^,^21^ Improvements in functioning and life circumstances are considered central features of recovery in many models, including AA, but assessment of these domains is a relatively recent development, primarily evident in clinical research.^18^,^21^ It is generally lacking in survey research that has provided the bulk of epidemiological data on population patterns of alcohol-related improvement, so this body of work only partially addresses the multiple domains considered important for investigating recovery, broadly defined.

A second core issue is that improvement in alcohol-related problems, including recovery from AUD, is a dynamic process of behavior change. Thus, longitudinal studies provide superior information to cross-sectional studies with retrospective assessments of drinking status, although the latter are common in the literature. Cross-sectional surveys have utility if they employ sound retrospective measures of past drinking status, but this is another qualification of the current epidemiological database on alcohol-related improvement and recovery. Longitudinal research has become more common in recent years. However, the intervals over which repeated measures are obtained rarely exceed 3 to 5 years, although there are notable exceptions with follow-ups of 8 to 10 years or more.^22^–^24^ Following large nationally representative samples for decades would be ideal, but the inevitable limitations on research resources have resulted in a collective body of work that generally comprises large representative studies that are cross-sectional or have short-term (e.g., 1 year) follow-ups. Studies with longer-term follow-ups tend to employ smaller, less representative samples. These core issues should be kept in mind when considering the epidemiology of improvements in alcohol-related problems, including recovery from AUD, as discussed next.

## RECOVERY PATHWAYS AND RELATIONSHIPS BETWEEN HELP-SEEKING AND DRINKING-RELATED OUTCOMES

Population-based survey research conducted over many decades has consistently revealed the following patterns with respect to improvements in alcohol-related problems:

- The majority of individuals who develop AUD reduce or resolve their problem over time.^7^,^8^,^25^ Rates of improvement vary widely depending on features of the research, such as the intervals over which drinking status was assessed (e.g., lifetime basis, shorter-term assessment based on a year or more); demographic characteristics, problem severity, and help-seeking status of respondents; and how improvement or recovery/remission was measured. But improvement over time is a reliable pattern and one that argues against a view of AUD as an inevitably progressive disease process.
- Seeking help for drinking problems from professional treatment or community and peer resources such as mutual help groups is uncommon,^1^,^26^ and a large gap persists between population need and service utilization. Most surveys indicate that less than 25% of persons in need utilize alcohol-focused helping resources.
- The great majority of persons who resolve their drinking problems do so without interventions, and such “natural recoveries” are the dominant pathway to problem resolution. Survey research has typically found that more than 70% of problem resolutions occur outside the context of treatment.^7^,^9^
- Stable low-risk drinking (moderation) is a relatively more common outcome in untreated samples, in part because seeking treatment is associated with higher problem severity,^7^,^12^ and most treatment programs emphasize abstinence.

For example, Fan and colleagues^7^ reported on the past-year prevalence of AUD recovery in the United States by using data from the NIAAA-funded 2012–2013 National Epidemiologic Survey on Alcohol and Related Conditions (NESARC-III)^2^ and DSM-5 diagnostic criteria.^3^ Survey respondents who met AUD criteria prior to the past year (*n* = 7,785) were assessed with respect to their current (past-year) AUD and risk drinking status. Drinking status was determined based on quantity-frequency criteria considered indicative of higher-risk drinking practices and DSM-5 AUD symptom counts. Measures of functioning and well-being were not collected.

Only 34% of respondents had persistent AUD, and most respondents had some degree of problem reduction; 16% achieved abstinence without symptoms, and 18% achieved low-risk drinking without symptoms. In addition, only 23% of the Fan et al. sample reported having ever received alcohol treatment, and those who did tended to fall into the persistent AUD (26%) or abstinent without symptoms (43%) outcome groups that generally are associated with higher problem severity.^7^ In contrast, among the subset of respondents who reported abstinence or low-risk drinking without symptoms, 87% of those who reported low-risk drinking without symptoms were never treated, and only 12% were treated. An additional 15% of the sample reported low-risk drinking with symptoms, and 15% reported high-risk drinking without symptoms.^7^ This is a refinement in outcome measurement compared to earlier surveys and illustrates the heterogeneity of recovery-relevant outcomes even in the absence of assessment of functioning and well-being.

This illustrative representative sample survey, among others,^8^,^9^ reveals a more optimistic and variable view of recovery pathways and outcomes than suggested by early research using treatment samples, which emphasized the chronic, relapsing nature of alcohol problems and the difficulty of maintaining remission. Population data indicate that, even though alcohol problems are prevalent, most affected individuals have less serious problems than the minority who seek treatment, and many improve on their own, including achieving stable abstinence or low-risk drinking without problems.

In contrast to these encouraging findings concerning rates of improvement, population research on the prevalence and patterns of help-seeking for alcohol-related problems indicates that the gap between need and service utilization is large and chronic. This is the case even though alcohol-related services have improved and expanded considerably over the past several decades^27^,^28^ and reliably yield benefits for a majority of recipients. Among the 25% or fewer who seek care, sources of care span the professional, community, and peer-helping sectors. Within the professional sector, care is diffused through mental health, medical, and community services systems, and only a minority receive alcohol-focused services from qualified programs or professionals.^8^,^27^

Prevalence estimates for utilization of different types of alcohol services are not reliably available for several reasons. For example, specialty treatment programs are often addiction-oriented and not alcohol-specific, most include mutual help group participation as a program requirement, and the anonymity principle of mutual help groups deters determination of utilization rates apart from treatment. Nevertheless, membership estimates for AA (2.1 million members worldwide, including 1.3 million U.S. residents; https://www.aa.org) suggest that AA participation is relatively widespread. Comparable membership data are not available for other mutual help groups such as Self-Management and Recovery Training (SMART Recovery), which holds more than 3,000 meetings per week worldwide (https://www.smartrecovery.org/), and LifeRing Secular Recovery, which offers more than 140 face-to-face meetings in the United States as well as online meetings and other electronic supports (https://www.lifering.org/). Regarding professional treatment, the 2016 National Survey on Drug Use and Health estimated that about 3.8 million U.S. residents age 12 and older received any type of substance use treatment in the past year,^27^ but these numbers are not specific to alcohol treatment. Also missing are data on relative remission rates as a function of type of care-seeking.

Higher problem severity predicts help-seeking, with higher severity reflected in greater alcohol dependence levels and alcohol-related impairment in areas of life functioning such as intimate, family, and social relationships; employment and finances; and legal affairs.^29^ Perceived need also predicts help-seeking; however, even among those who perceive a need, only 15% to 30% receive help,^30^ and problem recognition often precedes seeking care by a decade.^28^ Thus, although most individuals who develop AUD will eventually resolve their problem, treatment utilization remains less used as a pathway to recovery. This pattern has persisted for decades despite recent expansion in the spectrum of services beyond clinical treatment to offer less costly and less intensive services that often can be accessed outside of the health care system and are suitable for those with less severe problems.^28^ In addition, provisions of the Patient Protection and Affordable Care Act expanded access to and coverage of services for SUD.

## RECOVERY ACROSS THE LIFE SPAN

Studies that followed risk groups and people with drinking problems longitudinally—typically using smaller samples than survey research—provide information on patterns of improvement and recovery across the life span. Some studies assessed functioning and life circumstances, in addition to drinking practices, and revealed the following age-related patterns with respect to the onset of and improvements in alcohol-related problems:

- Drinking to intoxication, binge drinking, and alcohol-related problems increase during adolescence and early adulthood, generally peaking between ages 18 and 22. Prevalence of past-year binge drinking (45%) and AUD (19%) is highest in the early 20s^31^ and then decreases beginning in the mid-20s and continuing well after early adulthood. This nonlinear trajectory for the majority of adolescents and young adults, often termed “maturing out,” has been found in cross-sectional and longitudinal research using large national samples^2^,^32^,^33^ and by the annual cross-sectional National Survey on Drug Use and Health.^1^
- Adult role transitions (e.g., employment, marriage, parenthood) and personal maturation (e.g., decreased impulsivity) are associated with remission or recovery in early adulthood.^31^,^34^–^36^ As is the case for the general adult population with AUD, only about a quarter of adolescents and young adults in need of treatment receive it.^1^
- A subset of young adults who engage in harmful alcohol use and develop AUD in early adulthood show persistent or escalating problems in later life. Alcohol use before age 21 predicts persistence and severity of harmful use throughout the life span;^37^ however, reductions in problem drinking in early adulthood are more likely to occur among individuals who had the most severe problems at earlier ages.^34^
- Development of AUD is less common after age 25, and reductions in problem drinking, including recovery from AUD, continue past early adulthood and across the adult life span, including through late middle and old age (ages 60 to 80 and older).^22^,^34^ Reductions in problem drinking at older ages are predicted by relatively heavier alcohol use in early old age that prompted complaints from concerned others.^22^

These trends favoring increased remission rates over the life span are generally representative of the population, but can mask important nuances about age-related associations between problem onset, remission, and recurrence rates.^31^,^34^–^36^ For example, Vergés and colleagues^35^,^36^ used NESARC data from Waves 1 and 2 (from 2001–2002 to 2004–2005) to “deconstruct” age-related patterns of three different dynamic changes that contributed to overall age-related trends in the prevalence of DSM-IV alcohol dependence at each wave. Although rates of new alcohol problem onset and recurrence of or relapse to earlier problems declined with age, rates of persistence of alcohol problems over time were relatively stable across ages 18 to 50 and older. These different processes that contributed to the overall trend of decreased alcohol-related problems with increasing age suggest that “maturing out”—as young people assume adult roles—is not a sufficiently complete account of remission rates across the life span.

In related research that also used NESARC data from Waves 1 and 2, Lee and colleagues examined how rates of remission, which they termed “desistance,” from mild, moderate, or severe levels of AUD varied across age groups ranging between ages 20 to 24 and 48 to 55.^34^ Using Markov models to characterize patterns of longitudinal transitions in drinking status, they found differences in rates of AUD desistance from young adulthood to middle age as a function of AUD severity levels. Desistance rates from severe AUD, defined as six or more DSM-IV symptoms, were considerably higher in earlier age groups (ages 25 to 29 and 30 to 34) relative to older age groups (ages 35 to 39, 40 to 47, and 48 to 55) as compared to rates found in surveys that aggregated data across AUD severity levels. Desistance rates from moderate AUD showed a similar, but less dramatic pattern across age groups, whereas desistance rates from mild AUD were relatively stable across age groups. When considered with the work of Vergés and colleagues,^35^,^36^ these studies (1) show that resolution of severe AUD contributes heavily and distinctively to early adulthood remission prevalence, and (2) highlight the importance of deconstructing overall AUD prevalence curves by taking into account onset, remission, and recurrence of different levels of AUD severity over the life span.

Finally, a few studies observed increased binge drinking among middle-aged and older adults,^33^ suggesting dynamic changes may occur in binge drinking in midlife; these changes are not well researched. Similarly, most natural recovery research comprises samples showing that midlife recovery from AUD is normative.^9^,^38^ Middle age is also when treatment entry tends to occur.^5^ Recovery in midlife and later ages is associated with an accumulation of alcohol-related problems coupled with life contexts that support and reinforce maintenance of drinking reductions and involve post-resolution improvements in functioning and well-being.^38^,^39^

## ROLE OF GENDER AND RACE/ETHNICITY

### Remission

In addition to age, rates of recovery or remission of AUD symptoms vary by gender and race/ethnicity. Using NESARC Wave 1 data, Dawson et al. found that older age and female gender predicted abstinence, but not low-risk drinking, in both treated and untreated respondents who had alcohol dependence prior to the past year.^5^ Compared to non-Hispanic Whites, non-Hispanic Blacks had proportionately higher rates of abstinence than low-risk drinking. In the Fan et al.^7^ replication of Dawson et al.^5^ using NESARC-III data, female gender predicted both abstinence and low-risk drinking.

Also using NESARC-III data, Vasilenko et al. examined AUD prevalence by age and race/ethnicity (White, Black, Hispanic).^40^ Although AUD prevalence generally peaked in the 20s and declined steadily with age, prevalence was higher for Whites at younger ages and higher for Blacks at older ages. This cross-over pattern typically occurred around age 60. In midlife, prevalence was similar for Blacks and Whites. Also, Whites reported higher AUD rates than Hispanic respondents at all ages, and men reported higher AUD rates than women until older age, when women were more likely than men to report AUD in their 70s. However, the number of participants older than age 70 was very small.

The study by Lee et al. that investigated age-related patterns of AUD desistance as a function of AUD severity also found gender and race/ethnicity differences.^34^ Desistance patterns for males were generally consistent with the full sample findings—namely, elevated desistance rates for severe AUD in early adulthood and relatively stable rates for mild and moderate AUD. In contrast, females showed markedly higher rates of desistance from moderate AUD in early adulthood compared to older ages and attenuated rates of desistance from severe AUD compared to males during ages 30 to 34 only. With respect to race/ethnicity, results for Whites were generally consistent with the full sample, but findings differed for Hispanics and Blacks. For Hispanics, the early adulthood spike in rates of desistance from severe AUD was more time-limited, occurring only during ages 30 to 34 with much lower rates during ages 25 to 29. For Blacks, desistance rates for mild AUD also were relatively stable but were elevated for both moderate AUD (ages 25 to 29 and 30 to 34) and severe AUD (ages 25 to 29). For severe AUD, desistance rates among Blacks were very low during ages 30 to 34.

Patrick and colleagues analyzed age and gender relations with binge drinking using data from 27 cohorts of the annual Monitoring the Future surveys (1976 to 2004).^41^ Participants were followed from 12th grade (modal age 18) through modal age 29/30. Across cohorts, the age of peak binge drinking prevalence increased from age 20 in 1976–1985 to age 22 in 1996–2004 for women, and from age 21 in 1976–1985 to age 23 in 1996–2004 for men. Similar to the typical population life span trajectory for AUD remission, for men the high prevalence of binge drinking persisted through ages 25 to 26, followed by reductions during the late 20s. For women ages 21 to 30, more recent cohorts reported significantly higher binge drinking prevalence than in earlier cohorts, with risk remaining high throughout the 20s. These shifts toward older age of peak binge drinking prevalence indicate an extension of risks associated with harmful alcohol consumption in young adulthood, especially for women.

Taken together, these studies on rates of improvement by gender and race/ethnicity suggest that many of the differences observed involve variations in the timing and extent of reductions in binge drinking and AUD during either young adulthood or older age, even though all groups tended to show overall patterns similar to the population as a whole. Differences during midlife were less extensive, although this developmental period has not been the focus of much research.

### Help-Seeking

Help-seeking patterns and preferences also vary by gender and race/ethnicity. The gap between need and receipt of treatment is larger for women than for men, even after controlling for the higher prevalence of AUD and greater problem severity among men.^42^,^43^ For example, using NESARC data from Waves 1 and 2, Gilbert et al. found that women identified as having DSM-IV alcohol abuse or dependence at Wave 1 had significantly lower odds than men at Wave 2 of having used any alcohol service, specialty treatment, or mutual help groups.^42^ These utilization differences occurred even though women and men reported similar low perceived need for help and similar numbers of treatment barriers. Women were more likely to report expecting that their problem would improve without intervention, whereas men were more likely to report prior help-seeking that was unhelpful. No differences in service utilization or perceived need were found for race/ethnicity among White, Black, and Hispanic respondents. Consistent with the larger literature, greater alcohol problem severity was associated with higher odds of service utilization.

Studies using pooled data from multiple waves of the national probability samples collected in the National Alcohol Surveys found differences in service utilization as a function of gender and race/ethnicity.^44^,^45^ Zemore et al. used pooled data from three waves (1995–2005) to investigate lifetime alcohol treatment utilization and perceived barriers among Latinx respondents (*N* = 4,204).^44^ Among respondents, 3.4%, 2.7%, and 2.1% reported any lifetime treatment, AA participation, and institutional treatment, respectively. Men were significantly more likely than women to report receipt of any treatment services (5.6% vs. 1.1%), AA (4.7% vs. 0.6%), or institutional treatment (3.2% vs. 1.0%). Completion of the study interview in English (4.3%) versus Spanish (2.3%) also predicted higher utilization. These patterns were similar among the subsample of respondents who reported lifetime alcohol dependence, among whom rates of service utilization were much higher (20.4% for men and 15.3% for women). The authors suggested that underutilization of treatment by women and Spanish speakers may be due to cultural stigma against women with an alcohol problem, concerns about racial/ethnic stereotyping or stigmatization when seeking treatment, and additional barriers faced by individuals who are uncomfortable speaking English.

A later study using pooled data from the 2000–2010 National Alcohol Surveys included Whites, Blacks, and Latinx participants and found lower service utilization among Latinx, Blacks (vs. Whites), and women (vs. men).^45^ Racial/ethnic differences in utilization were moderated by gender. Among women, only 2.5% of Latinas and 3.4% of Blacks with lifetime AUD used specialty treatment compared to 6.7% of Whites; among men, the corresponding figures were 6.8% for Latinos, 12.2% for Blacks, and 10.1% for Whites.^45^ Higher utilization among Whites than among Blacks and Hispanics also was found using the 2014 cohort from the National Survey on Drug Use and Health.^46^

Overall, research on race/ethnicity and help-seeking is not extensive, and groups other than Whites, Blacks, and Hispanics/Latinx have not been well studied.^47^ Available research suggests that the gap between need and service utilization common among those with an alcohol problem is accentuated among ethnic and racial minority groups; however, research is in its infancy on why this is the case and how to address it.

## DISCUSSION

Research on the epidemiology of recovery from AUD is somewhat uneven in scope and methods, and gaps remain in the knowledge base. Nonetheless, the bulk of evidence converges in showing that (1) improvements in alcohol-related problems, including recovery from AUD, are commonplace; (2) natural recovery is the dominant pathway; (3) greater problem severity is associated with treatment utilization; and (4) low-risk drinking outcomes are more common among untreated samples. Problem prevalence and rates of remission of AUD symptoms in the U.S. population peak during the 20s and are followed by a slow, steady decline over the adult life span. The specific ages when these characteristic dynamics in the temporal patterning of harmful alcohol use and remission of symptoms occur vary somewhat as a function of gender and race/ethnicity, but the overall general pattern is well established.

These findings provide a rich foundation concerning population patterns and dynamics of recovery, remission, and help-seeking. Future research aimed at disaggregating these complex associations at the population level should be a priority and can inform approaches to promoting remission and recovery in two general ways.^48^ First, longitudinal studies of the onset of and improvements in alcohol-related problems^31^,^34^–^36^ exemplify how epidemiological risk factors are reliably associated with the course of alcohol problem development and improvement and can be used to target at-risk individuals for preventive interventions. Second, “upstream” population-level interventions can be applied to prevent or reduce the determinants of risk (e.g., through changes in policy, taxation, and health and community infrastructure). The latter approach, although less common, takes advantage of the well-established prevention paradox—small reductions in harmful alcohol use by risky drinkers with less serious problems result in far greater health improvements at the population level than do changes in harmful alcohol use by the minority of persons with AUD.

This body of research qualifies the usual characterization of AUD as a chronic, relapsing/remitting disorder for which intensive intervention is essential for recovery. That characterization may be representative for a small minority of persons with more severe AUD, but it is inaccurate for the large majority of persons with mild to moderate problems, many of whom resolve their problems the first time they attempt to quit and often without interventions.^9^,^49^ Whether this qualification applies to SUD other than AUD is not established.

The recovery literature is characterized by a mix of cross-sectional population surveys with short-term retrospective assessments (1 year is typical) and prospective follow-ups of smaller-sized samples of risk groups that, with some notable exceptions,^22^–^24^ also had relatively short follow-ups. Use of data from the multiple waves of the NESARC dominates this research literature. Although the NESARC obtained data from a very large nationally representative sample of the U.S. population age 18 and older (e.g., *N* = 36,309 in NESARC-III), it shares limitations inherent to most survey research—namely, assessments must be relatively brief, meaning that the domains of inquiry must be limited and selected carefully and cannot be probed to obtain the detail typically useful in clinical applications.

These design characteristics have contributed to gaps in the literature due to overreliance on drinking practices as the major outcome metric and less common measurement of functioning, well-being, and life circumstances, which are central features of recovery and can occur with or without reductions in drinking. Correlates of remission rates are being reported with increasing frequency in survey research, but tend to be limited to demographic characteristics, problem severity variables related to drinking practices, help-seeking history, and, in some cases, psychiatric comorbidity. Other than the seminal research program of Moos and colleagues,^22^,^39^ assessment of functioning, context, and well-being surrounding drinking behavior change is a relatively recent development, primarily evident in clinical research^18^,^21^ and process-oriented research on natural recovery.^38^ Connecting these research literatures in meaningful ways in future investigations is essential for broadening scientific knowledge about how affected individuals reduce and resolve their alcohol-related problems and for guiding improvements in alcohol services that are responsive to heterogeneity in recovery-related outcomes and pathways.

Another issue in need of further research involves deconstruction of separable processes that contribute to overall problem prevalence and remission rates across the life span. As highlighted in the research of Vergés, Lee, Sher, and colleagues,^31^,^34^–^36^ overall population rates are influenced by age-related associations between problem onset, remission, and recurrence rates, which raises questions about whether remission patterns reflect a simple “maturing out” of harmful alcohol use that began in early adulthood. Based on the available data, Lee and Sher^31^ concluded: “[T]he continual declines in AUD rates observed throughout the life span . . . appear mainly attributable to reductions in new onsets . . . whereas potential for desistance from an existing AUD may peak in young adulthood . . . [especially] for those with a severe AUD” (p. 37).

The timing and targeting of prevention and treatment programs could be refined to enhance intervention effectiveness if these age-related associations between problem onset, remission, and recurrence rates were firmly established and used to guide intervention delivery. Conducting this kind of research is challenging because it requires collecting data on all three processes over the life span, and there are additional complexities in studying the tails of the age distribution. For example, clinical diagnostic systems may overdiagnose AUD in adolescence, which would inflate estimates of remission rates in early adulthood.^50^ Attrition biases are of concern with advancing age as poor health and death may remove proportionately more older adults with AUD from population samples, thereby inflating estimates of remission rates in old age particularly from severe AUD.^5^,^34^

A final generalization from this research concerns the limited contribution of alcohol treatment or other alcohol-focused services to recovery prevalence in the population. Low rates of service utilization have persisted despite improvements in AUD treatment and lower threshold options^28^ and the expansion of access and coverage of services for SUD provided by the Affordable Care Act. The enduring gap between population need and service utilization despite these advances strongly suggests that alternative avenues are needed to increase intervention diffusion and uptake. It has proven insufficient to offer improved treatment predominately through the health care sector, and priority needs to be given to reaching broader segments of the at-risk population of drinkers who contribute most of the alcohol-related harm and cost. Nevertheless, a sizable subset of individuals with AUD improve or recover without interventions, and recent evidence suggests that individuals with more severe AUD exercise some degree of appropriate self-selection into treatment.^29^ Empirical questions warranting further investigation are how to distinguish among individuals or risk groups for whom natural recovery is a high probability outcome and how to segment the market so that treatment services are targeted and available for those in need who are not likely to achieve recovery without treatment.

Further improvements in reducing the prevalence of AUD and increasing the prevalence of recovery likely depend on dissolving the silos that have long existed between clinical and epidemiological research and applications^11^ and finding novel ways to disseminate evidence-based services to the large underserved at-risk population of drinkers who will not use professional services, at least in their present form. It is also important to consider a broader public health approach to dispel long-held beliefs that alcohol is a problem only for those with severe AUD and that those with AUD can resolve their problem only through abstinence. Perpetuation of these myths over many decades has stigmatized the disorder and deterred help-seeking among the millions of people who would benefit from drinking reductions.

In conclusion, recovery from AUD and alcohol-related problems is the most common outcome among those with problem alcohol use, and recovery without abstinence is possible, even among those with severe AUD. Changing the narrative to highlight the high likelihood of recovery could help engage more individuals in alcohol-related services and may encourage individuals to reduce their drinking in the absence of formal treatment.
